# Immunofluorescence Staining of Paraffin Sections Step by Step

**DOI:** 10.3389/fnana.2020.582218

**Published:** 2020-11-09

**Authors:** Sami Zaqout, Lena-Luise Becker, Angela M. Kaindl

**Affiliations:** ^1^Basic Medical Science Department, College of Medicine, QU Health, Qatar University, Doha, Qatar; ^2^Charité – Universitätsmedizin Berlin, Institute of Cell and Neurobiology, Berlin, Germany; ^3^Charité – Universitätsmedizin Berlin, Center for Chronically Sick Children (Sozialpädiatrisches Zentrum, SPZ), Berlin, Germany; ^4^Charité – Universitätsmedizin Berlin, Department of Pediatric Neurology, Berlin, Germany

**Keywords:** immunofluorescence, paraffin sections, antibodies, microscopy, imaging

## Abstract

Immunofluorescence staining is the most frequently applied technique to detect and visualize various molecules in biological samples. Many protocols can be found in the literature and the websites of commercial antibody producers. This can result in a time-consuming and costly methodical work to establish “simple” antibody staining. We here summarize in a stepwise manner an easy-to-follow immunofluorescence staining protocol with an improved specific fluorescent signal and a reduced background and non-specific binding signal. This will help scientists to save time, effort, and antibody costs during the application of such a valuable technique.

## Introduction

Immunofluorescence (IF) staining is a method of choice in studying the subcellular localization of proteins in fixed biological samples (Zaglia et al., 2016; Niedenberger and Geyer, 2018; Smith and Gabriel, 2018). It provides researchers with an easy tool to detect and compare the distribution of proteins in the cells and tissues of various model organisms. It relies on antibody/antigen-specific binding, and it has direct and indirect approaches (Becheva et al., 2018). In the case of direct immunostaining, the primary antibody against the antigen of interest is directly conjugated to a fluorophore that enables direct fluorescent detection using a fluorescent microscope. In the indirect immunostaining approach, a fluorophore-conjugated secondary antibody against the unconjugated primary antibody is applied (Becheva et al., 2018; Niedenberger and Geyer, 2018). This method can be performed on cultured cells (immunocytochemistry, ICC) and tissues (immunohistochemistry, IHC) (Maity et al., 2013). IHC can be applied on tissues prepared either through paraffin embedding or through cryopreservation.

Despite its wide application, inexperienced researchers often face trouble in successfully detecting and analyzing a clear fluorescent signal of their protein of interest. Doubts behind unsuccessful or poor quality staining usually revolve around antibody quality and efficiency. This is time-consuming and cost-intensive, with a frequent search for alternative antibodies. The aim of our present paper was to provide researchers, especially those who just started using the IF method, with a detailed stepwise protocol in order to achieve a high success rate, strong specific fluorescent signals, and negligible unspecific and/or background signals. This, in turn, will allow them to easily identify and analyze their protein of interest. Following this protocol will also help researchers to obtain high-quality fluorescent images to be used for their final representative publication images.

## Materials and Methods

### Mice and Tissue Preparation

All experiments were carried out in accordance with the national ethics principles (registration no. T0309.09). Organs from postnatal 0 (P0) and 6- to 12-week-old adult *C57BL/6* mice were obtained from the animal facility of the Charité—Universitätsmedizin Berlin, Germany.

After dissection, the tissues used in this study were fixed in 4% paraformaldehyde (PFA) in 0.12 M phosphate buffer (TPO_4_) overnight (adult brains), for 4 h (adult testes and skeletal muscles), or for 10 min (P0 eyes) at 4°C. The thick capsule of adult testes was punctured at the beginning with a needle to allow rapid penetration of the fixative (Zaqout et al., 2017). Tissues were then dehydrated in an ethanol series, cleared with xylene, and embedded in paraffin, as described previously (Canene-Adams, 2013; Sadeghipour and Babaheidarian, 2019). Five- to ten-micrometers sections were finally cut on a microtome and collected on SuperFrost Plus^®^ slides (J1800AMNZ, Thermo Fisher Scientific Inc., Germany).

### General Instructions Before Starting

(a) General lab safety guidelines in the workplace (e.g., lab coat, gloves, mask, and working under a fume hood) should be followed.
(b) The time and type of fixation should be taken into consideration during tissue preparation, especially with some antibodies that are extremely fixation-sensitive (Ramos-Vara, 2005; Shi et al., 2007).
(c) If applicable, antibodies already used in previously published articles should be targeted first. The quality of the presented IF staining should be critically reviewed in terms of signal-to-noise ratio.
(d) Antibodies directed toward the protein of interest can be searched in the websites of commercial antibody producers/companies or in the resources of updated scientific products such as Biocompare^1^.
(e) In the case of using a newly produced commercial or homemade antibody, validation experiments should be performed to determine their specificity, selectivity, and reproducibility (Hsi, 2001; Shi et al., 2007; Saper, 2009; Bordeaux et al., 2010). Western blot, blocking peptides, and negative controls are the commonly used validation methods (Skliris et al., 2009; Bordeaux et al., 2010). In order to confirm the antibody’s specificity, it is always recommended to use positive and negative controls based on experimental settings and lab facilities (for reviews, see Hsi, 2001; Shi et al., 2007; Saper, 2009; Bordeaux et al., 2010; Holmseth et al., 2012; Magaki et al., 2019).
(f) The storage conditions and the expected expiry dates of the antibodies, as usually stated in the accompanying antibody sheets, should be cross-checked and noted.
(g) The following protocol mainly describes IHC on paraffin-embedded tissues; however, the dewaxing and heat antigen retrieval steps can be skipped in order to be applicable on cryopreserved tissues.
(h) Most of the following experimental steps can be performed at regular room temperature (RT); however, incubation at 4°C is better for some antibodies, in case recommended by the antibody producer/company. This, if wished, can be tested at the beginning by incubating two samples at both temperature conditions.
(i) Fresh distilled water (d-H_2_O) is used to prepare the following solutions and dilutions.

### Preparation of Solutions

#### Solutions for the Dewaxing Step

For the dewaxing step, about 300 ml of the following solutions is needed to fill each of the histological staining boxes accordingly, as will be described later:

(a) Two glass bottles of each 100, 95, and 70% ethanol series (C_2_H_6_O; K928.4, Carl Roth GmbH, Germany)
(b) Three glass bottles of xylene (C_8_H_10_; 9713.3, Carl Roth GmbH)
(c) One glass bottle of xylene/ethanol (1:1)

These solutions can be prepared in advance and stored in tightly closed glass bottles at RT for a long time. They can also be reused several times and need to be replaced only when they turn dirty.

#### Solution for the Heat Antigen Retrieval (Unmasking) Step

The unmasking solution is prepared by mixing 3.6 ml of a citrate-based antigen retrieval solution (pH 6.0; H-3300, Vector Laboratories, United States) with 400 ml d-H_2_O. The pH should be closely monitored as some antigens need different pH ranges from 3 to 10; however, for most of them, a pH of 6.0 seems to be satisfactory (Boenisch, 2005; Ramos-Vara, 2005). The unmasking solution is prepared freshly on the first day of the experiment and can be used only for one batch of paraffin slides (e.g., 10 slides or 20 slides back to back). In the case of having more slides, additional unmasking solution should be prepared.

#### Solutions for the Rinsing and Permeabilization Steps

For rinsing, 1,000 ml of phosphate-buffered saline (PBS) 1X is prepared by mixing 100 ml of PBS 10X (14190-250, Thermo Fisher Scientific Inc.) with 900 ml d-H_2_O. This solution can be prepared in advance and stored at RT. It is recommended to use this solution within 1 month.

For permeabilization, a PBS 1X/gelatin (0.2% *w*/*v*)/Triton (0.25% *v*/*v*) solution is prepared as follows, which needs to be stored at 4°C and is best used within 10 days:

(a) Using a 1,000-ml cylinder, 500 ml of PBS 1X is prepared as described above.
(b) Using a 500-ml glass beaker, 500 ml d-H_2_O with 2 g of gelatin (1040781000, Merck KGaA, Germany) is heated (up to 55°C) until completely dissolved.
(c) The dissolved gelatin solution is then added to PBS 1X (prepared in step 1).
(d) After the solution has cooled down, 2.5 ml Triton 100X (9002931; Sigma-Aldrich, Germany) is added slowly due to its sticky nature.
(e) The final solution is then mixed using plastic-coated magnetic stir bars.
(f) The solution is then filtered with a filter paper (240 mm; 4.303.240, Neolab, Germany) into a 1,000-ml glass bottle (pH 7.4) (Gandhi and Khare, 2018).

#### Solution for the Blocking Step

Five percent bovine serum albumin (BSA) is prepared by dissolving 0.25 g BSA (A 9647, Sigma-Aldrich) in 5 ml permeabilization solution (pH 7.4; for its preparation, see section “Solutions for the Rinsing and Permeabilization Steps”). This solution will be used to block non-specific binding and has to be prepared freshly and stored at 4°C for use within 48 h. It can, in addition, be diluted to 1% in order to be used for primary and secondary antibody dilutions.

#### Solutions for the Primary and Secondary Antibody Incubation Steps

Generally, the primary antibodies can be divided into monoclonal and polyclonal groups, with the monoclonal ones only binding to a single epitope, thereby being more specific, and the polyclonal ones, which bind to multiple epitopes, being more sensitive (Magaki et al., 2019). To avoid cross-reactivity that can occur if the antibody has a high affinity toward a different antigen with a similar structural region (Ivell et al., 2014), it is recommended to use a primary antibody that has been raised against a different species from the animal model that it is applied to Ramos-Vara (2005); Manning et al. (2012), and Ivell et al. (2014). For example, a rabbit primary antibody would be a good choice for application on mouse cells or tissues. For double or triple IF, the primary antibodies need to be raised in different species in order to be detected individually using two or three secondary antibodies of different fluorescence detection wavelengths (e.g., green, 488 nm; red, 555 nm; far red, 633 nm). In fact, antibodies with blue or far-red fluorophores are either less easily or not detected by the human eye, respectively, compared to those with green–red fluorophores (Marshall and Johnsen, 2017). In some cases, directly conjugated primary antibodies can be used to skip the step of using separate secondary antibodies.

An ice box can be used to keep the antibodies and dilutions cold. The desired concentrations of the primary and secondary antibodies are prepared in 1% BSA (see section “Solution for the Blocking Step”). Primary antibody solutions are prepared on the first day of the experiment during the blocking step (section “Dewaxing Step”), while the secondary antibody solutions are prepared on the second day during the second rinsing step (section “Second Rinsing Step”). For the first trial, preferably three different concentrations of a primary antibody should be tested, and these should include the dilution recommended by the producer or in the literature as well as concentrations above and below the recommended dilution (Magaki et al., 2019). For example, if a dilution of 1:300 is recommended, a scientist could test 1:100, 1:300, and 1:500. For secondary antibodies, it is similarly recommend to test multiple concentrations with the lowest background and still optimal signal (Hoffman et al., 2016). In our hands, 1:400 is the best concentration that works for most secondary antibodies. In order to detect nuclei signals, 40,6-diamidino-2-phenylindole (DAPI, 1 μl/ml; D9542, Sigma-Aldrich) is added to the secondary antibody solution. The volume of these concentrations is calculated according to the number of paraffin microscopic slides to be stained. Usually, 200 μl of the antibody dilution per slide is sufficient, however, it is better to calculate one more slide in order to avoid not having enough of the required volume for the last slide. For example, for the antibody solution for four slides, prepare five times 200 μl = 1,000 μl solution. In the case of double or triple IF, the solution of two or three primary antibodies, respectively, is prepared in the same 1% BSA solution on the first day of the experiment. It needs to be ensured that matching secondary antibodies with different fluorescence detection wavelengths are available. The solution of these matching secondary antibodies together with DAPI is also prepared in the same 1% BSA solution on the second day of the experiment.

The antibodies used for the IF experiments in this paper are well-established and were previously applied by our group and by others on mouse tissue (Table 1). In addition, negative control staining experiments including the application of only the secondary antibody have been performed (data not shown).

**TABLE 1:** List of the primary and secondary antibodies used in this study.

Name	Source	Catalog no.	RRID no.	Dilution
Rabbit anti-NeuN	Merck-Millipore, Germany	ABN78	AB_10807945	1:200
Mouse anti-tubulin beta III Isoform (Tuj1)	Merck-Millipore, Germany	MAB1637	AB_2210524	1:3000
Rabbit anti-SOX2	Abcam, Cambridge, United Kingdom	ab59776	AB_945584	1:500
Rabbit anti-mouse vasa homolog (MVH/DDX4)	Abcam, Cambridge, United Kingdom	ab13840	AB_443012	
Rabbit anti-laminin	Abcam, Cambridge, United Kingdom	ab11575	AB_298179	1:1000
Mouse anti-phospho-histone H3 (pH3)	Cell Signaling Technology, Germany	9706	AB_331748	1:100
Chicken anti-Pax7	Hybridoma Bank, Iowa, United States	AB_528428	AB_528428	1:200
Mouse anti-parvalbumin (PV)	Swant, Switzerland	PV 235		1:2000
Donkey Cy3-conjugated anti-rabbit	Jackson ImmunoResearch, Suffolk, United Kingdom	711-165-152		1:400
Goat Alexa Fluor^®^ 488-conjugated anti-mouse (IgG)	Invitrogen, Darmstadt, Germany	A-21121		
Goat Alexa Fluor^®^ 488-conjugated anti-chicken (IgG)	Invitrogen, Darmstadt, Germany	A-11039		
Goat Alexa Fluor^®^ 633-conjugated anti-rat (IgG)	Invitrogen, Darmstadt, Germany	A-21094		
Goat Alexa Fluor^®^ 633-conjugated anti-mouse (IgG)	Invitrogen, Darmstadt, Germany	A-21052		
Goat Alexa Fluor^®^ 405-conjugated anti-mouse (IgG)	Abcam, Cambridge, United Kingdom	ab175671		
				

#### Solution for the Final Rinsing Step

In addition to PBS 1X (see section “Solutions for the Rinsing and Permeabilization Steps”), 10 mM copper (II) sulfate (CuSO_4_)/50 mM ammonium chloride (NH_4_Cl) solution is prepared as follows: 0.8 g of CuSO_4_ (*MW* = 159.61; 451657-50G, Sigma-Aldrich) and 1.3 g NH_4_Cl (*MW* = 53.49; A9434, Sigma-Aldrich) are dissolved together in 500 ml d-H_2_O using plastic-coated magnetic stir bars. This solution reduces lipofuscin-like autofluorescence, which can complicate the detection of specific IF signals (Partanen et al., 1980; Schnell et al., 1999). The bottle containing this solution needs to be covered with aluminum foil and kept in the dark at RT. It is recommended to use this solution within 1 month.

### Stepwise Procedures

#### First Day

##### Dewaxing Step

**FIGURE 1:**
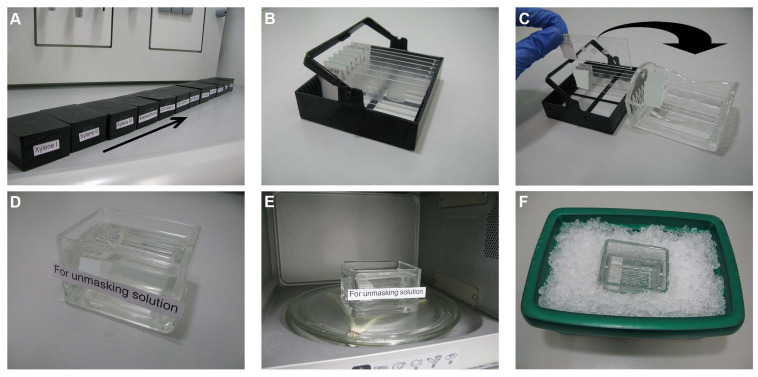
Dewaxing and heat antigen retrieval steps. **(A)** Twelve histological staining boxes are cleaned, labeled accordingly, and filled with the related solutions. **(B)** The paraffin section slides are distributed in staining racks. **(C)** After dewaxing, the slides are transferred to glass staining racks and then to glass staining containers **(D)** filled with the unmasking solution. **(E)** The container with the slides is microwaved for 3 min after boiling, cooled down to approximately 50°C using an ice box **(F)** and then microwaved again for 3 min.

Twelve histological staining boxes (2285.1, Carl Roth GmbH) are cleaned, labeled accordingly, and filled with the related solutions prepared in a previous step (section “Solutions for the Rinsing and Permeabilization Steps”) and dH_2_O as follows (Figure 1A):

(a) Boxes 1–3 for xylene I–III
(b) Box 4 for xylene/ethanol
(c) Boxes 5 and 6 for 100% ethanol I and II
(d) Boxes 7 and 8 for 95% ethanol I and II
(e) Boxes 9 and 10 for 70% ethanol I and II
(f) Boxes 11 and 12 for d-H_2_O I and II

These boxes are then closed with lids to avoid evaporation and kept under a fume hood. The paraffin slides of the target tissue are selected under a bright-field microscope and labeled with the name, species, and the concentration of the tested antibody/antibodies. The paraffin section slides are distributed in staining racks (2285.1, Carl Roth GmbH) (Figure 1B). These racks will be incubated in the aforementioned boxes as follows:

a. Xylene I–III for 15 min each
b. Xylene/ethanol for 5 min
c. 100% ethanol I and II for 2 min each
d. 95% ethanol I and II for 2 min each
e. 70% ethanol I and II for 2 min each
f. d-H_2_O I and II, twice for 2 min each

The sections can be kept in d-H_2_O for up to 2 h.

##### Heat Antigen Retrieval (Unmasking) Step

The unmasking solution, prepared in a previous step [section “Solution for the Heat Antigen Retrieval (Unmasking) Step”], is poured in a glass-staining container (H554.1, Carl Roth GmbH) or in an empty microwave-proof 1,000-μl pipette tip container. Dewaxed slides are transferred to glass staining racks (10193482, Thermo Fisher Scientific Inc.) (Figure 1C) and placed in the unmasking solution (Figure 1D). The container with the slides is microwaved as follows (Figure 1E):

a. The container with the slides is microwaved at 800 W for 8 min until the unmasking solution starts to boil.
b. From the moment the solution starts to boil, the container should remain in the microwave for further boiling for 3 min. Microwave incubation time should be increased proportionally in the case that thicker sections are used (Shi et al., 2007).
c. The container and its contents are then carefully transferred to an ice box (Figure 1F), where it should cool down to approximately 50°C (takes about 10–15 min). Use a thermometer to measure the temperature of the solution.
d. The container with the slides is boiled again for 3 min.
e. The container with its contents is then carefully transferred to an ice box again, where it should cool down to RT (takes about 30–45 min).

The sections can be kept in the unmasking solution for up to 2 h.

##### Initial Rinsing and Permeabilization Steps

**FIGURE 2:**
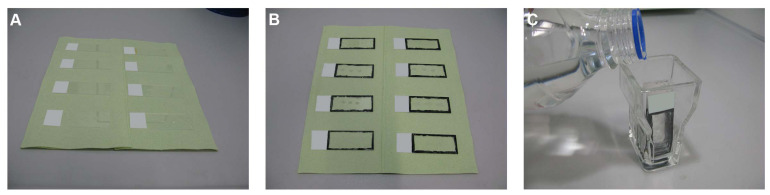
Initial rinsing and permeabilization steps. **(A)** The slides are taken out from the unmasking solution and placed on tissue papers. **(B)** The rim of each slide is marked using a hydrophobic barrier pen or a nail polish. **(C)** The sections are then placed in glass cuvettes and rinsed as described in the text.

The slides are then taken out and placed on tissue papers, making sure that the sides with the sections face upwards (Figure 2A). The sections are kept this way for about 1 min until they are semidry; drops of the solution can be removed using a paper towel. The rim of each slide (alternatively, the region around one, several, or all sections) is marked using a hydrophobic barrier pen (PAP pen; ab2601, Abcam, Cambridge, United Kingdom) or a nail polish (Figure 2B). The sections are then placed in glass cuvettes (Shandon^TM^ vertical staining jar; 140, Thermo Fisher Scientific Inc.) (Figure 2C) and rinsed as follows:

a. PBS, twice for 2 min each
b. PBS for 10 min
c. PBS/gelatin/Triton 0.25%, twice for 10 min each

##### Blocking Step

**FIGURE 3:**
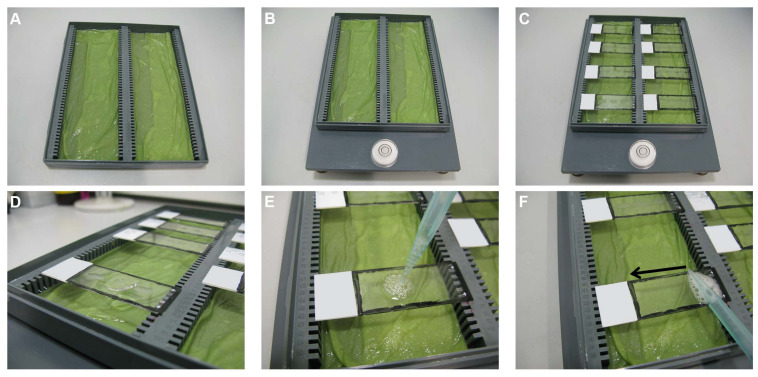
Blocking step. **(A)** A microscope slide box is used to prepare a humid box by wetting tissue papers and placing them at the bottom of the microscopic slide box grooves. **(B)** The box is placed on a flat balanced surface. **(C)** The slides are placed horizontally, one next to the other, on the edges of the humid box. **(D)** Using a 1,000 μl pipette, 190 μl of 5% BSA is added to each slide and the pipette is slightly pressed more to create some air bubbles **(E)** on top of the solution. **(F)** The air bubbles are then touched with the side of a pipette tip and slid over the slides to distribute the solution above the sections evenly without touching the sections.

A microscope slide box is used to prepare a humid box by wetting tissue papers and placing them at the bottom of the microscopic slide box grooves (Figure 3A). The purpose of this is to avoid the evaporation of the added solutions during the incubation periods. It has to be ensured that the box is placed on a flat balanced surface (e.g., leveling plate with bubble spirit) (Figure 3B). It is also recommended to keep the box away from sources of vibration and from direct sunlight exposure.

The slides are taken out of the PBS/gelatin/Triton 0.25% solution and placed horizontally one next to the other (without touching each other) on the edges of the humid box (Figure 3C). Using a 1,000-μl pipette, 190 μl of 5% BSA (section “Solution for the Blocking Step”) is added per slide (Figure 3D) and the pipette is slightly pressed more to create some air bubbles (Figure 3E) on top of the solution. These air bubbles are then touched with the side of a pipette tip and slid over the slides to distribute the solution above the sections evenly without touching the sections (Figure 3F). The humid box is closed carefully by its lid and kept for 60 min at RT.

##### Incubation With Primary Antibody Step

**FIGURE 4:**
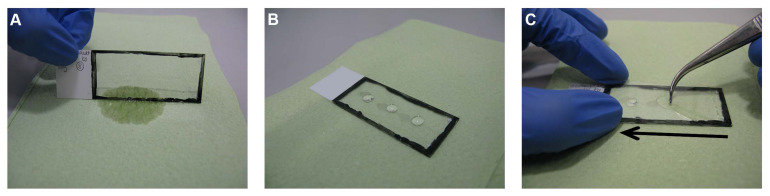
Mounting step. **(A)** The slides are taken out and the extra solution is dispelled on tissue paper. **(B)** Three separate drops of Immu-Mount^TM^ are added on the slide. **(C)** The slides are then covered with cover glass starting from one side of the slide and quickly pressed to the other side by applying light pressure guided by a forceps to avoid air bubbles.

The slides are taken out from the humid box and the blocking solution is dispelled on tissue papers (Figure 4A). The slides are then placed back into the humid box as described above. Of the primary antibody solution (section “Solutions for the Primary and Secondary Antibody Incubation Steps”), 190 μl is added per slide and distributed evenly, as described in section “Blocking Step” (Figure 3F). Care should be taken to adding each antibody solution to the correct matching labeled slide. The humid box is closed carefully by its lid and kept overnight at RT on a flat balanced surface without agitation.

#### Second Day

##### Second Rinsing Step

The slides are taken out from the humid box and the primary antibody solution is dispelled on tissue paper (Figure 4A). Using the glass cuvettes (see section “Initial Rinsing and Permeabilization Steps”), the sections are rinsed as follows:

a. PBS, twice for 10 min
b. PBS/gelatin/Triton 0.25% for 10 min

The sections are then again placed back in the humid box. Of the secondary antibody solution (section “Solutions for the Primary and Secondary Antibody Incubation Steps”), 190 μl is added to each slide and distributed evenly, as described in section “Blocking Step” (Figure 3F).

##### Final Rinsing and Mounting Steps

The slides are taken out from the humid box and the secondary antibody solution is dispelled on tissue paper (Figure 4A). Using the glass cuvettes (see section “Initial Rinsing and Permeabilization Steps”), the sections are rinsed as follows:

a. PBS, three times for 10 min
b. 10 mM CuSO_4_/50 mM NH_4_Cl solution (section “Solution for the Final Rinsing Step”) for 10 min
c. Short rinse with dH_2_O

The slides are then taken out and placed on tissue papers, making sure that the section sides are facing upwards (Figure 2B). Once the sections have dried (approximately 3 min), three separate drops of Shandon^TM^ Immu-Mount^TM^ (10662815, Thermo Fisher Scientific Inc.) are added on the slide (Figure 4B). The slides are then covered with a coverslip starting from one end of the slide and quickly pressed to the other end, guided by forceps to avoid air bubbles (Figure 4C). The slides are then kept in a horizontal position for drying in the dark for 24 h at RT before imaging. For longer storage, the slides can be sealed with nail polish and subsequently stored in slide boxes in the dark at 4°C. If imaging rapidly is needed, the slides can be imaged directly after mounting without using oil (no higher magnification).

#### Imaging Step

It is recommended, as mentioned above, to take images of the IF slides 24 h after mounting to allow sufficient hardening of the mounting medium. In this study, fluorescent images were taken with an Olympus BX51 microscope by an Intas camera and Magnafire 2.1B software (Olympus, Hamburg, Germany) or by an lsm5exciter Zeiss confocal microscope with the software Zen (version 2009, Zeiss, Jena, Germany). All images were processed using Adobe Photoshop CS6 version 13.0 × 64.

## Results and Discussion

Although the IF method has been used for a long time, various efforts to improve the results have been undertaken and some of them published (Ramos-Vara, 2005; Kim et al., 2016; Nasr et al., 2018). We here aimed to achieve a synthesis of several of these ideas and added practical details that have been proven useful in our hands. In a stepwise manner, we describe the IF protocol for paraffin sections and have incorporated many years of personal experience in using this technique on various tissues. This will expand the protocols described by other groups that either focus on a specific tissue type or lack practical step-by-step information and photos (Mason et al., 2000; Ramos-Vara, 2005; Gandhi and Khare, 2018; Magaki et al., 2019).

**FIGURE 5:**
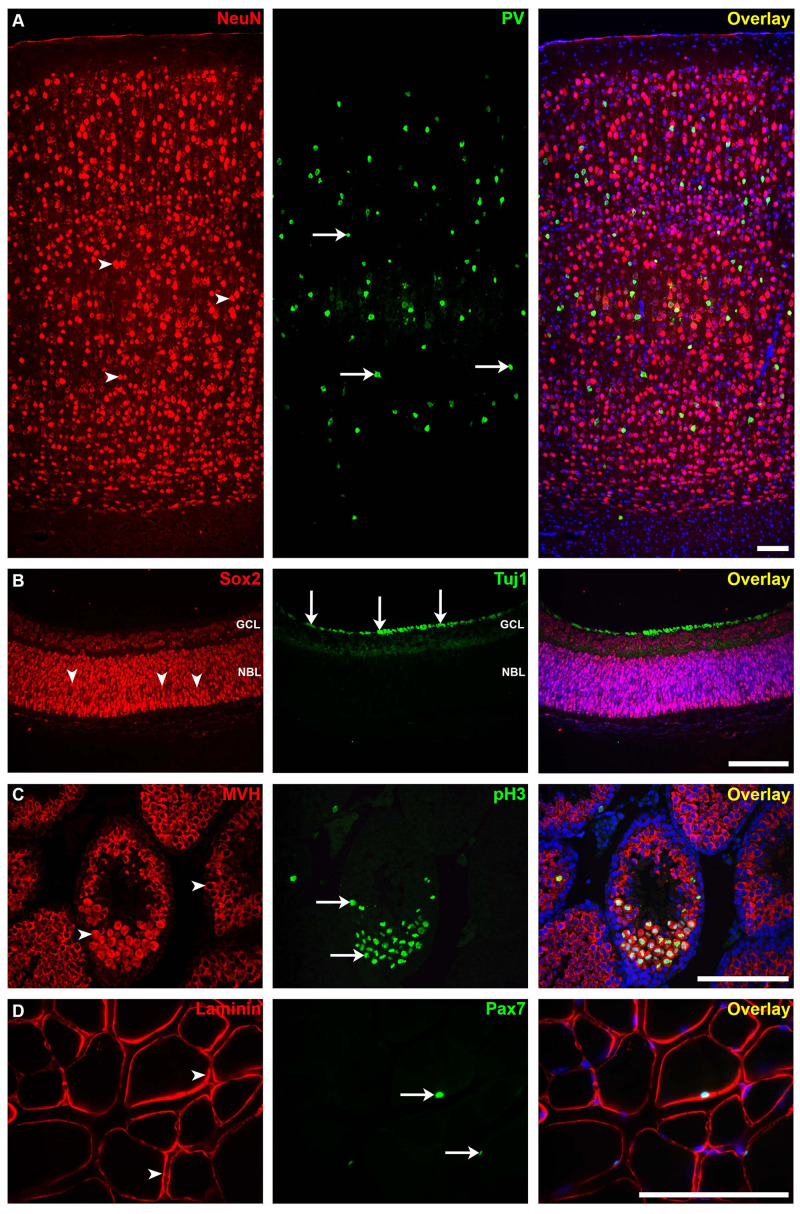
Immunofluorescence staining for different mouse tissues using our protocol. **(A)** The somatosensory neocortical region of an adult brain section stained for neuronal nuclei (*NeuN*; mature neuron marker, *arrowheads*) and parvalbumin (*PV*; interneuron marker, *arrows*). **(B)** Postnatal 0 (P0) retina section stained for sex-determining region Y—box 2 (*SOX2*; stem cell marker, *arrowheads*) and anti-tubulin beta III isoform (*Tuj1*; early neuron marker, *arrows*). *GCL*, ganglion cell layer; *NBL*, neuroblastic layer. **(C)** Adult testis section showing seminiferous tubules stained for mouse vasa homolog (*MVH*; spermatogenic cell marker, *arrowheads*) and anti-phospho-histone H3 (*pH3*; mitotic cell marker, *arrows*). **(D)** Adult skeletal muscle section stained for laminin (basement membrane marker) and paired box protein-7 (*PAX7*; muscle precursor cell marker, *arrows*). Immunofluorescence images: *scale bar*, 100 μm. For more details on the antibodies used in this figure, please refer to Table 1.

Using this protocol, we were able to detect and study strong IF signals in mouse brain, retina, testis, and muscle (Figures 5A–D, respectively). The technique can be applied for other tissue types, both paraffin-embedded sections and cryosections. However, we believe, as also others (Niedenberger and Geyer, 2018), that the tissue quality and cellular morphology are much well-preserved in paraffin-embedded tissues. In addition, it has been reported that non-specific background staining is less common for paraffin-embedded tissue sections than for frozen sections (Larsson, 1988; Elias, 2003). Although mice have been used in our experiments, this protocol can also be applied on tissues from other species.

Being one of the most common problems in IF, the intensity of non-specific background staining is affected by multiple factors. Following recommendations available in the literature (Oliver and Jamur, 1999; Schnell et al., 1999; Boenisch, 2001; Ramos-Vara, 2005), we used proper fixation, dewaxing, antigen retrieval, blocking and permeabilization agents, and conditions that minimize these factors. Using suitable antibody concentrations, adequate washing, and adding a quenching solution to the last wash were also crucial determinants in achieving the best quality in our hands.

Formaldehyde is the most widely used fixative in IHC because it preserves the general structure of cellular organelles (Boenisch, 2001; Ramos-Vara, 2005). Care should be taken particularly with respect to the fixation time and temperature, which in turn can affect the cross-link formation. Both over- and under-fixation can produce false-negative or false-positive results (Oliver and Jamur, 1999; Ramos-Vara, 2005). In addition, formalin over-fixation increases the hydrophobicity of proteins, which in turn increases background staining (Ramos-Vara, 2005). Superior to glutaraldehyde, the effects of formaldehyde over-fixation can be partially reversed (Eltoum et al., 2001). Moreover, using a proper retrieval method on formalin-fixed tissue sections resulted in optimal immunostaining levels regardless of the fixation time (Shi et al., 1998, 2007). The tissues shown in this study were fixed in 4% PFA at 4°C for a time period ranging from 10 min (P0 eyes) via 4 h (adult testes and skeletal muscles) to overnight (adult brains). We achieved the best staining result of the testicular seminiferous tubules using this fixative and after puncturing the thick capsule of the testes (Figure 5C; Zaqout et al., 2017). The testes can be alternatively fixed in a mixture of 3.7% formalin with 0.2% glutaraldehyde and 0.05% saponin (Benson and Busch, 1996) or in Bouin solution (Ellenburg et al., 2020). A wide range of other fixatives can be used for other tissue types and for special antibody staining (for reviews, see Hopwood, 1969; Arnold et al., 1996; Oliver and Jamur, 1999; Eltoum et al., 2001). The applied fixative and buffer solutions can be of high importance in scientific projects addressing cellular reactions to, e.g., ischemic conditions such as microglia morphology changes (Cătălin et al., 2017).

Dewaxing steps have been described slightly differently by other protocols e.g., incubating the paraffin sections in xylene I–III for 5–15 min each, skipping the incubation in xylene/ethanol or adding a step of 50% ethanol for 5 min (Gandhi and Khare, 2018; Magaki et al., 2019). In our lab, we never obtained altered outcomes after changing these steps.

The first step after dewaxing, the antigen retrieval, renders the tissue more accessible to the subsequent antibody binding, thereby increasing the sensitivity of IHC (Boenisch, 2001; Shi et al., 2007; Scalia et al., 2017; Magaki et al., 2019). The majority of formalin-fixed tissues require this important step to optimize the immunoreaction (Ramos-Vara and Beissenherz, 2000). Most commonly, a combination of chemical treatment and heat [heat-induced epitope retrieval (HIER) via microwave] is used to break protein cross-links caused by fixation. Both 10 mmol/L citrate buffer (pH 6.0) and 1 mmol/L EDTA buffer (pH 8.0) can be applied in HIER treatment, especially when a new antibody is tested (Hsi, 2001). Using 10 mmol/L citrate buffer (pH 6.0), with heating at 97°C for 20–60 min, has been proven optimal staining for various antibodies (Boenisch, 2005; Ramos-Vara, 2005; Shi et al., 2007). In our protocol, we used HIER where the slides are microwaved at 800 W for 8 min until the citrate-based unmasking solution starts to boil, followed by 3 min of boiling, cooling down to 50°C, and again microwaving for an additional 3 min to then finally cool the solution down to RT. Other protocols use different microwave wattages, different boiling/cooling-down times, or a reduced temperature peak of only 65°C with only 240 W (Zaglia et al., 2016; Gandhi and Khare, 2018; Magaki et al., 2019). In general, a high temperature for a short period (10 min) is preferred over a low temperature for a long period (Hayat, 2002). Less commonly, protease-induced epitope retrieval (PIER) has been used as an enzymatic antigen retrieval method (Ramos-Vara and Beissenherz, 2000). However, this method can alter the tissue morphology and destroy epitopes (Ordonez et al., 1988). Other antigen retrieval protocols include different physical treatments like ultrasound and other applications to deliver heat, such as pressure cookers and water baths (Magaki et al., 2019).

It has been reported that the buffer used for the preparation of the rinsing and permeabilization solutions as well as the primary and secondary antibody solutions can affect the preservation of antigenicity (Elias, 2003). We have used PBS 1X (pH 7.2–7.5) continuously without notable side effects. Usually, antigen–antibody reactions in the case of polyclonal antibodies are not altered by slight changes in the buffer pH, unlike reactions with monoclonal antibodies (Absolom and van Oss, 1986). The choice of a specific buffer can also be influenced by the objective and the antibody used. For example, Tris buffer normal saline (TBS) is recommended for alkaline phosphatase procedures (Elias, 2003).

The 0.25% PBS/gelatin/Triton solution applied in our protocol is used to permeabilize the tissue and increase the immunostaining efficiency. It has been found that rinsing the sections in such Triton concentration yielded better results than when using 0.5 or 1% Triton alone (Gandhi and Khare, 2018). In addition, higher Triton concentrations can affect the preservation of tissue morphology and antigenicity, while lower concentrations are usually insufficient to achieve homogeneous immunostaining (Lawrence and Golubeva, 2017).

The blocking step is applied in order to minimize potential non-specific antibody binding generated by hydrophobic, ionic, hydrogen, and other intermolecular interactions. Blocking with 5–10% normal serum is commonly used to prevent non-specific antibody binding governed by ionic and hydrophobic interactions (Daneshtalab et al., 2010; Buchwalow et al., 2011). In this case, researchers should use the serum matching the species of the secondary antibody in use and, if multiple secondary antibodies are used, trying the sera from both species if the background signal is high (Buchwalow et al., 2011; Magaki et al., 2019). Alternatively, blocking with BSA is found to prevent non-specific binding and background by blocking the hydrophobic interaction between proteins and ionic or electrostatic interactions (Kim et al., 2003; Buchwalow et al., 2011). There are also multiple commercial blocking agents, and others suggested a combined use of 10% horse serum and 1% BSA (Gandhi and Khare, 2018). Indeed, we achieved less background noise using 5% BSA for 60 min at RT as a blocking agent compared to 10% normal goat or donkey sera. In addition, BSA is much more economical compared to normal serum since only a very small amount of highly concentrated proteins is used in the preparation of the blocking solution. In some protocols, sections are placed in 3% hydrogen peroxide for up to 5 min and washed afterward with deionized water for 5 min before the heating step to block endogenous peroxidase activity (Radulescu and Boenisch, 2007; Magaki et al., 2019). Intriguingly, others have not reported differences in the staining results and claimed that the blocking step is not necessary (Buchwalow et al., 2011).

Autofluorescence generated by the accumulation of lipofuscin pigments in various tissue types can affect the detection of specific IF signals dramatically (Partanen et al., 1980; Schnell et al., 1999). In line with the recommendation of others, we found that the use of copper sulfate with ammonium chloride in the final rinsing step helped in quenching the autofluorescence effect frequently seen in paraffin-embedded tissues (Schnell et al., 1999). Other protocols described the use of Sudan Black B in ethanol or other reagents to reduce the autofluorescence signals (Sun et al., 2011; Gandhi and Khare, 2018). However, Sudan Black B is incompatible with some xylene-based permanent mounting media (Schnell et al., 1999), and it reduces both specific and non-specific signal intensities drastically (Gandhi and Khare, 2018). In the case that highly vascularized tissues as well as frozen brain sections are used, a series combination of sodium borohydride, crystal violet, and Sudan Black B leads to total quenching of autofluorescence background, masking of lipofuscin, and non-specific signals (Gandhi and Khare, 2018).

In summary, we provide researchers, especially those inexperienced, an easy-to-follow stepwise IF protocol that will help them achieve a high specific fluorescence signal and a reduced background binding. This will in turn save effort, cost, and time in performing this technique.

## Data Availability Statement

The raw data supporting the conclusions of this article will be made available by the authors, without undue reservation.

## Ethics Statement

The animal study was reviewed and approved by the animal facility of the Charité – Universitätsmedizin Berlin, Germany. All experiments were carried out in accordance to the national ethic principles (registration no. T0309.09).

## Author Contributions

SZ and AMK were responsible for the project conception. SZ and L-LB performed the experiments. SZ wrote the manuscript. All authors read, revised, and approved the final manuscript.

## Conflict of Interest

The authors declare that the research was conducted in the absence of any commercial or financial relationships that could be construed as a potential conflict of interest.

## Footnotes

1 https://www.biocompare.com/
